# Heterogeneous Morphologies and Hardness of Co-Sputtered Thin Films of Concentrated Cu-Mo-W Alloys

**DOI:** 10.3390/nano14181513

**Published:** 2024-09-18

**Authors:** Forrest Wissuchek, Benjamin K. Derby, Amit Misra

**Affiliations:** 1 Department of Material Science and Engineering, University of Michigan, Ann Arbor, MI 48109, USA; fwissu@umich.edu; 2 Center for Integrated Nanotechnologies, Los Alamos National Laboratory, Los Alamos, NM 87545, USA; benderby@lanl.gov

**Keywords:** heterogeneous microstructures, nanocomposite, physical vapor deposition, nanoindentation

## Abstract

Heterogeneous microstructures in Cu-Mo-W alloy thin films formed by magnetron co-sputtering immiscible elements with concentrated compositions are characterized using scanning transmission electron microscopy (STEM) and nanoindentation. In this work, we modified the phase separated structure of a Cu-Mo immiscible system by adding W, which impedes surface diffusion during film growth. The heterogeneous microstructures in the Cu-Mo-W ternary system exhibited bicontinuous matrices and agglomerates composed of Mo(W)-rich phase. This is unique, as these are the slower-diffusing species, contrasting past reports of binary Cu-Mo thin films that exhibited Cu-rich agglomerates. The bicontinuous matrices comprised of Cu-rich and Mo(W)-rich phases exhibited bilayer thicknesses of less than 5 nm. The hardness of these thin films measured using nanoindentation is reported and compared to similar multilayers and nanocomposites in binary systems.

## 1. Introduction

Establishing accurate processing-structure relationships for co-sputtered immiscible thin films plays an important role in advancing the design of multiphase and heterogeneous materials. The kinetic conditions during physical vapor deposition (PVD) cause the immiscible elements to phase separate and self-assemble into microstructures comprised of periodic concentration modulations (CMs) of different orientations and periodicity; far from equilibrium conditions cause metastable phases and heterogeneities to arise. Heterogeneous materials can offer superior combinations of strength and ductility compared to monolithic materials because of unique interactions between co-deforming phases [1,2,3,4,5].

The effects of deposition rate, temperature, and phase fraction on the resulting multiphase morphology of binary systems containing an FCC transition metal and a BCC refractory metal have been established through phase field modeling and experimental validation. The effect that the choice of BCC refractory metal plays on the resultant morphology, however, has not been systematically explored, even though differences in the melting point can greatly affect the kinetics of phase separation at a given processing temperature [6,7,8,9].

For physical vapor deposition (PVD) processes, diffusion at the growing surface dominates the overall kinetic process because it occurs at a greater rate than bulk diffusion, particularly at relatively lower homologous temperatures [8]. The homologous temperature ($T_{h}$):

(1) $$T_{h}=\frac{T_{deposition}}{T_{melting}}$$

describes how close the processing temperature, in [K], is to an element’s melting point, and it is a useful kinetic factor for relating processing temperature to structure. Surface diffusivity increases monotonically with temperature, with a rapid increase as the processing temperature nears the melting point of a constituent element [7].

Besides $T_{h}$, deposition rate (v) is another key kinetic factor that influences the phase separated morphology. During PVD, the surface is continually buried by the incoming deposition flux, which retards bulk diffusivity. Higher deposition rates result in less time for an adatom to diffuse on the surface [8,9]. The effects of $T_{h}$ and v on phase separation and the resulting morphology can be combined into one kinetic term, the surface interdiffusion length, ρ, which represents the distance an adatom can travel on the surface before it is buried by the deposition flux. The surface interdiffusion length is expressed as

(2) $$\rho =\sqrt{\frac{D_{s}\delta }{v}}$$

where *D_s_* is the surface interdiffusivity, δ is the thickness of the surface layer, and v is the deposition rate [8].

Studies on the effects of the homologous temperature and deposition rate on phase separated thin films with equiatomic compositions classify morphologies into vertical (VCM), lateral (LCM), and random (RCM) concentration modulations [6]. VCMs and LCMs are phase separated layers that run parallel and perpendicular to the substrate, respectively [6,9]. Monomodal phase separated morphologies can be fully described by one of these classifications and the bilayer wavelength of the CM. Increasing ρ leads to larger bilayer wavelengths, as the adatoms diffuse further on the surface before being buried [8]. Increasing ρ also leads to a transition from VCM to LCM morphologies in binary systems with near equiatomic compositions [6,9]. The formation of an LCM morphology is attributed to complete phase separation at the surface, as opposed to VCM morphologies, which form due to incomplete phase separation at the surface and are a driving force for phase separation for atoms buried in the bulk [9].

For an immiscible system containing an FCC transition metal and a BCC refractory metal, there is a large difference in the homologous temperature ($∆T_{h})$ of each constituent element at a given processing temperature. For example, the homologous temperatures of Cu, Mo, and W at a substrate temperature of 400 °C are 0.50, 0.23, and 0.18, respectively. At 800 °C, they are 0.79, 0.37, and 0.29, respectively. $∆T_{h}$, as well as the difference in surface diffusion length (∆ρ) between the FCC and BCC components, is maximized as the processing temperature approaches the melting point of the FCC component [7].

When $∆T_{h}$ and ∆ρ are large, the disparity between the FCC and the BCC adatom mobility can lead to the formation of heterogeneous morphologies. Compared to monomodal morphologies, which are fully described by the orientation and bilayer wavelength of the CM, heterogeneous morphologies have structural features that span multiple length scales and require more complex descriptions. For example, Derby et al. reported a Cu-Mo thin film with a layered Cu-Mo matrix and Cu agglomerates [10]. The Cu agglomerates contained superlattices of Mo nanoprecipitates. In the matrix, the Cu and Mo layers had coherent interfaces such that the Cu layers adopted a metastable BCC crystal structure. The Cu agglomerates had the equilibrium FCC structure and the Mo nanoprecipitates adopted a non-equilibrium FCC structure to maintain coherency with the surrounding Cu agglomerate.

The heterogeneous morphology reported by Derby et al. formed because Cu adatoms are more mobile on the surface, as indicated by their larger $T_{h}$ and ρ relative to Mo [10]. Cu adatoms can travel larger distances over the surface before being buried, so they form agglomerates with sizes proportional to their surface diffusion length [7]. Any slower-diffusing atoms of BCC refractory metal become trapped within the FCC Cu agglomerate and self-organize into arrays of nanoprecipitates due to their negligible solid solubility. The matrix surrounding the Cu agglomerates phase separates to form a CM. Phase field modeling by Powers et al. found that local fluctuations in the composition of the vapor flux help to promote heterogeneous phase separation [11]. Phase field simulation discovered that if a localized region begins with a composition disparity of ≈70 Cu–30 Mo at.% or greater, the minority phase forms discrete nanoparticles rather than lamellar concentration modulations in order to minimize surface energy. This structure is like that of an agglomerate with embedded nanoprecipitates.

While using $T_{h}$ as a measure of temperature helps to generalize the results of phase field models to immiscible systems of any composition, the phase field models do not take $∆T_{h}$ into account for predicting morphology [9]. Thus, it is unclear if the models for heterogeneous structure formation or for transitions between homogeneous morphologies are accurate for systems with larger $∆T_{h}$ values.

Phase separated thin films with tunable CM orientations and heterogeneous morphologies give rise to unique mechanical properties as compared to metallic multilayers, which are restricted to VCM morphology with monomodal CMs. In metallic multilayers, refining layer thickness increases strength because interphase boundaries are barriers to dislocation transmission [12,13]. The strongest multilayers have layer thicknesses of less than 10 nm, at which point dislocation pileup can no longer occur. Instead, deformation occurs via confined layer slip (CLS)—the extension of a single dislocation loop along the interphase boundary [13]. As layer thickness is further reduced to below ≈ 5 nm, the CLS stress may far exceed the characteristic barrier stress for single dislocation transmission across the interface, resulting in localized shear bands and the loss of uniform elongation. Co-sputtered thin films allow small scale deformation mechanics to be studied on a wider variety of morphologies. Furthermore, co-sputtered immiscible thin films, particularly those with heterogeneous morphologies, are more resistant to shear banding failure than traditional multilayers [12]. Highly aligned slip systems in multilayers allow for the easy propagation of dislocations through many layers. The intertwined interfaces in co-sputtered thin films misalign slip systems and suppress shear bands. Additionally, in heterogeneous morphologies with soft phase agglomerates, the agglomerates deform first and then strain harden until they reach the hardness of the surrounding matrix. The heterogeneous material can undergo significant plastic deformation while maintaining the hardness of a fine multilayer. Cui et al. compared the deformation behavior of Cu-Mo nanocomposites using micropillar compression [12]. A multilayer with a 3 nm layer thickness had a maximum flow stress of 2.8 GPa, but failed at only 2.5% plastic strain. The heterogeneous microstructure, with an average layer thickness of 3–4 nm and included Cu agglomerates, had a maximum flow stress of 2.6 GPa and deformed plastically, strain hardening until it reached 12% plastic strain. Bulk alloys with heterogeneous structures at micrometer scales have also been reported to have superior combinations of strength and plasticity, and hetero-deformation induced (HDI) strengthening is proposed to be a strengthening mechanism that relies on the interactions between soft zones and ultrafine-grained hard zones to achieve high rates of strain hardening [1,2,3].

Studies of co-sputtered immiscible alloy thin films thus far have focused on understanding the effects of deposition temperature, deposition rate, and phase fraction on binary systems. The microstructure’s evolution and the resulting mechanical behavior of ternary (and higher) alloy systems is unexplored. Ternary systems offer the ability to finely tune differences in surface diffusivity and control the resulting morphology. To study how composition effects structure through changes in diffusivity, a material system that does not introduce additional phases is ideal. For this purpose, the Cu-Mo-W system was chosen. The Cu-Mo-W system is univariant—the phase fraction only changes due to changes in the Cu concentration because Mo and W are completely soluble with one another. For any composition at equilibrium, two phases are present: an FCC phase composed of Cu and a BCC phase composed of a Mo-W solid solution. Therefore, this system can easily be compared to a binary Cu-Mo system and used to study how composition changes the kinetics of phase separation.

## 2. Materials and Methods

### 2.1. Thin Film Fabrication by Co-Sputtering

The thin films were deposited by direct current (DC) magnetron co-sputtering in a Kurt J Lesker PVD 75 deposition chamber. The 2-inch diameter Cu, Mo, and W targets, of 99.999%, 99.95%, and 99.95% purity, respectively, were arranged confocally with a 30° angle between the substrate normal and the vapor flux and a 12.7 cm throw distance. The substrates were (100) Si thermally oxidized with a 1000 nm SiO_2_ surface layer. The substrates were mounted on a heated stage, and the three samples were heated to 400, 600, and 800 °C and rotated at 20 rpm for the duration of each deposition. The base pressures prior to deposition were below 8 × 10^−5^ Pa. The process gas used was Ar with a flow rate of 30 sccm, and the working pressure was 0.4 Pa. Prior to the deposition, the substrate was sputter cleaned with an RF bias of 30 W for 30 min, and a 10 W RF bias was applied during the deposition. The deposition duration was 5500 s. The Cu, Mo, and W target powers were 125, 70, and 85 W, respectively, with corresponding deposition rates of about 0.11, 0.09, and 0.08 nm/s, respectively. The samples were slowly cooled under vacuum when the deposition finished.

### 2.2. Chemical and Crystallographic Characterization

X-ray florescence (XRF) was performed with a Rigaku Supermini 200 (Rigaku Americas Corporation, Woodlands, TX, USA). Scans were performed on 6 identical samples from the same deposition, measuring about 1 cm^2^ each, to maximize the signal. The X-ray generator voltage was 50 kV, and the current was 4 mA. X-ray diffraction (XRD) was performed using a Rigaku SmartLab (Rigaku Americas Corporation, Woodlands, TX, USA) with a Cu Kα (λ = 1.5406 Å) radiation source. The X-ray generator voltage was 40 kV, and the current was 50 mA. A grazing incidence X-ray diffraction (GIXRD) geometry with a 1 degree incidence angle was used.

### 2.3. Microstructural Characterization

Foils were prepared for scanning transmission electron microscopy (STEM) using focused ion beam (FIB) milling with a Thermo Fisher Scientific (TFS) Helios 650 Nanolab (TFS, Hillsboro, OR, USA) dual beam SEM/FIB. A TFS Talos F200X G2 S/TEM (TFS, Hillsboro, OR, USA) was used for STEM and STEM energy dispersive X-ray spectroscopy (STEM-EDS) characterization. The accelerating voltage was 200 keV and aSuper-X window-less detector system (Bruker, Billerica, MA, USA) was used for STEM-EDS. A TFS Spectra 300 Probe-Corrected S/TEM (TFS, Hillsboro, OR, USA) operated at 300 keV accelerating voltage was used for high-resolution STEM.

### 2.4. Nanoindentation

Nanoindentation was performed using a Hysitron 950 TriboIndenter (Bruker, Eden Prairie, MN, USA) with a standard Berkovich probe. The tip area function of the probe was calibrated on fused quartz. Indents were load controlled with a peak load of 1 mN. Indentation depths were 150–300 nm and each film’s thickness was about 1.5 µm. A grid containing 9 indents spaced 20 µm apart was performed on each sample. SEM images of the indents were taken with a TFS Helios 650 Nanolab SEM to measure the indent area and calculate hardness.

## 3. Results

### 3.1. Composition

Cu-Mo-W thin films were co-sputtered onto substrates heated to 400 °C, 600 °C, and 800 °C. The corresponding $T_{h}$ values for Cu are 0.50, 0.64, and 0.79, for Mo are 0.23, 0.30, and 0.37, and for W are 0.18, 0.24, and 0.29. The combined deposition rate for each film was 0.28 nm/s, 0.28 nm/s, and 0.27 nm/s, respectively. This was measured by dividing the film thickness, measured by cross-section STEM, by the duration of the deposition. Deposition conditions were chosen to promote heterogeneous structure formation; elevated substrate temperatures and low deposition rates maximize ∆ρ. In addition to the processing temperature and deposition rate, phase field models have also studied the effect of phase fraction on the resulting microstructure. At compositions outside of ~0.4 < *c_o_* < ~0.6, where *c_o_* is the phase fraction of one phase, the minority phase forms discrete particles rather than monomodal CMs [9,14]. A similar trend was expected for a univariant ternary system, so samples with Cu composition between 40–60 at.% were prepared. The compositions measured qualitatively by XRF are reported in Table 1. The samples had 40, 41, and 47 at.% Cu respectively, for increasing substrate temperature.

### 3.2. Effect of Far from Equilibrium Processing on Global Crystal Structure

**Figure 1:**
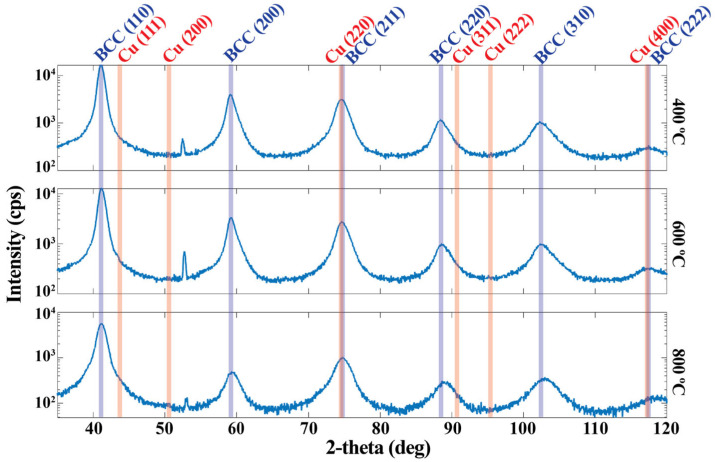
2-theta X-ray diffractograms with a 1° incidence angle for Cu-Mo-W films co-sputtered at 400 °C, 600 °C and 800 °C. Calculated peak positions for BCC Mo and W are shown in blue and for FCC Cu in red. The measured Bragg peaks match the calculated BCC peaks.

X-ray diffractograms of the samples deposited at 400 °C, 600 °C, and 800 °C are presented in Figure 1. The vertical blue lines represent the calculated peak positions for Mo and W, which have equilibrium BCC structures with 3.142 Å and 3.155 Å lattice parameters, respectively [15]. The vertical red lines represent the calculated peak positions for Cu, which has an equilibrium FCC structure with a 3.597 Å lattice parameter [15].

The measured Bragg peaks correspond to a BCC structure with a lattice parameter of 3.10 Å. The small peak at 52.4 degrees does not match the expected peak locations for FCC Cu or BCC Mo or W and is assumed to arise from the oxide layer of the Si substrate or from the XRD stage. The lack of Bragg peaks from an FCC phase suggests that even though the Cu-Mo-W mixture decomposes to have Cu-rich domains, on average these domains do not adopt the bulk equilibrium FCC structure and instead adopt the BCC structure of the surrounding layers. This is a phenomenon that has been reported in the early stages of spinodal decomposition [16,17] and in multilayers with small layer thicknesses [18]. When Cu takes the BCC structure, it forms coherent interfaces with the surrounding layers, which can reduce the surface energy enough to overcome the increased volume strain energy at small layer thicknesses. The STEM observations discussed in the next section confirm that the samples contain a coherent nanograined matrix.

### 3.3. Heterogeneous Morphology with Mo-W Agglomerates and a Random Bicontinuous Matrix

**Figure 2:**
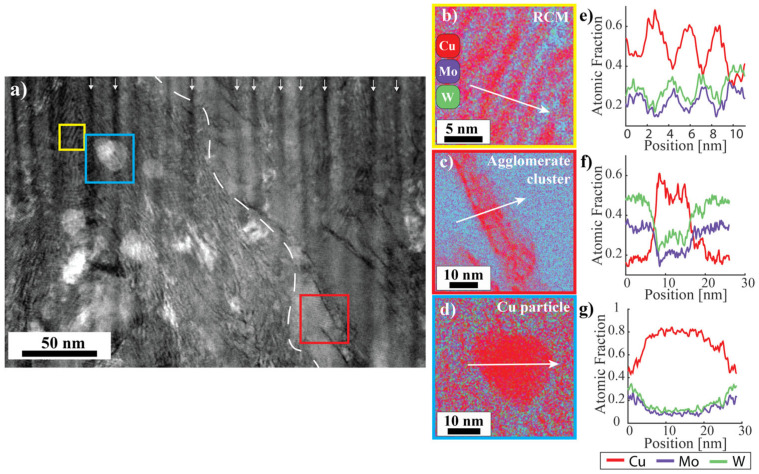
(**a**) A cross-section HAADF micrograph of the Cu-Mo-W sample deposited at 400 °C. The white dashed line marks the division between the RCM and the agglomerate cluster and the colored boxes correspond to the locations of the STEM-EDS micrographs of (**b**) the periodic concentration modulation, (**c**) a Mo-W agglomerate cluster, and (**d**) a spherical Cu agglomerate. (**e**–**g**) Composition line profiles matching the three features listed in (**b**–**d**), respectively. The position axes correspond to the white arrows in (**b**–**d**).

High-angle annular dark-field STEM (HAADF-STEM) and STEM energy dispersive X-ray spectroscopy (STEM-EDS) of the sample deposited at 400 °C are shown in Figure 2. The region to the left of the dashed line depicts the bicontinuous matrix, which is made evident by the alternating light and dark contrast in HAADF. The region to the right of the dashed line depicts clusters of Mo-W agglomerates where there is no periodic concentration modulation.

An example of the RCM is enclosed by a yellow box in Figure 2a, and the corresponding EDS map and line profiles in Figure 2b,e reveal that the RCM consists of alternating Cu-rich and Mo-W rich layers with a bilayer thickness of about 3 nm. The dark vertical lines in Figure 2a marked by white arrows are curtaining artifacts from focused ion beam (FIB) sample preparation and do not represent a microstructural feature. The RCM is interrupted by clusters of agglomerates, which appear as large regions of nearly uniform contrast. The red box in Figure 2a contains a section of the agglomerate cluster; the corresponding EDS map and line profile appear in Figure 2c,f. The agglomerates are a Mo-W solid solution phase, and Cu veins intersect the agglomerates. The Cu veins tend to maintain a similar orientation across a given agglomerate cluster. These clusters span regions on the order of 100 nm in diameter, but Cu agglomerates segment the Mo-W solid solution into smaller regions, and the Cu veins are less than 10 nm thick.

The micrographs of the bicontinuous matrix of the 400 °C sample contain spherical agglomerates with bright HAADF contrast. One such agglomerate is marked by a blue box in Figure 2a. The agglomerates are 20–30 nm in diameter. EDS analysis shows that they contain a high concentration of Cu, as seen in the EDS map and line profiles in Figure 2d,g. Bright HAADF contrast is typically indicative of elements with a high atomic number, but diffraction contrast can also contribute to HAADF signal.

**Figure 3:**
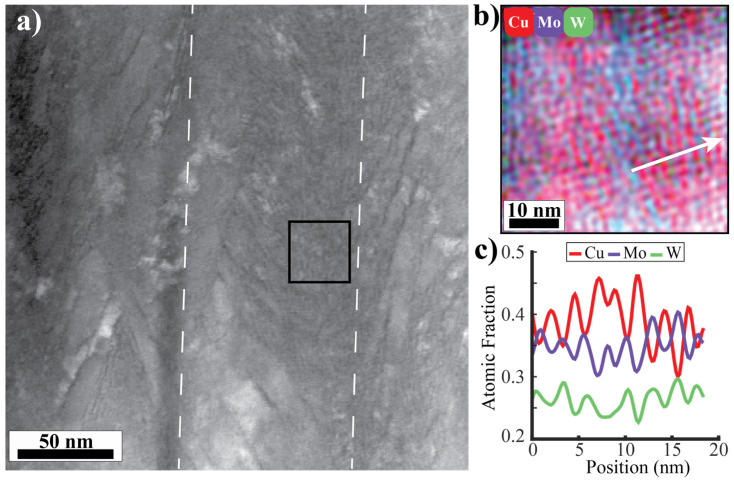
(**a**) HAADF-STEM of the 600 °C sample. The dashed lines mark the locations of columnar grain boundaries. The black box indicates the location from which the EDS was collected. (**b**) A STEM-EDS micrograph of the VCM. The white arrow corresponds to the (**c**) line profile showing the wavelength of the CM.

The sample deposited at 600 °C contains the same primary features as the 400 °C sample: a bicontinuous Cu/Mo-W matrix and Mo-W agglomerates. Figure 3a shows HAADF-STEM of the sample. The average intercolumnar diameter is approximately 50 nm. The Cu veins in the Mo-W agglomerate clusters gradually transition into the bicontinuous matrix by broadening and slowly incorporating more Mo-W. The STEM-EDS and composition profile in Figure 3b,c, taken from the center of the bicontinuous matrix and marked by a white arrow in Figure 3b, show a region with an individual layer thickness of approximately 1.8 nm. The bilayer wavelength is approximately 3 nm on average.

The micrographs of the sample deposited at 600 °C do not exhibit any of the spherical Cu agglomerates that appeared in the STEM images from the 400 °C sample. Additionally, while both samples had a bicontinuous matrix and Mo-W agglomerate clusters, the distribution of these features was different at each processing temperature. These regions were columnar and had widths of about 50 nm in the 600 °C sample, but they did not show any specific organization in the 400 °C sample. While these differences are important to note, for the purpose of identifying transitions between structural zones as a function of temperature in this system, these features do not denote a large enough difference to consider different kinetic formation mechanisms.

**Figure 4:**
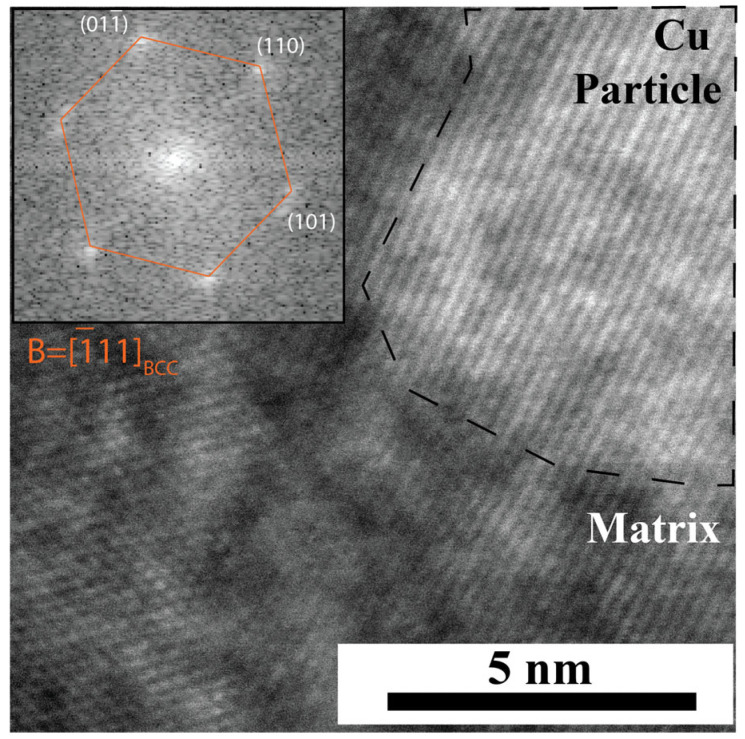
HRSTEM micrograph of the 400 °C sample. The matrix in the upper left corner is aligned with the BCC $\left[\bar{1}11\right]$ zone axis. The spherical Cu agglomerate in the upper right is aligned with a low symmetry zone axis.

For both samples, the layers of the concentration modulation were coherent. Cu adopted the BCC structure of its surroundings rather than its FCC bulk equilibrium structure. Figure 4 shows part of the coherent BCC matrix and a spherical Cu agglomerate in the 400 °C sample. The high symmetry zone axis seen in the bottom left of the micrograph corresponds to the matrix that contains alternating Cu and Mo-W layers. The corresponding FFT in the upper left is of the BCC $\left[\bar{1}11\right]$ zone axis. The Cu layers have a pseudomorphic BCC structure and are coherent with the Mo-W layers. A Cu agglomerate is outlined in the upper right corner of the micrograph. The matrix and the agglomerate could not be aligned with the high symmetry zone axis simultaneously. However, the spacing of the fringes in the Cu agglomerate was measured to be 2.35 ± 0.10 Å. The calculated spacing of (110) planes in a BCC W lattice with a 3.17 Å lattice parameter and (111) planes in an FCC lattice with 3.62 Å lattice parameter are 2.24 Å and 2.09 Å, respectively. The measured lattice fringes from the agglomerate match better with the BCC (110) planes. The lattice parameter is not an exact match for BCC Mo/W, and the orientation of the BCC Cu agglomerate does not exactly match the surrounding matrix, as evidenced by the change in zone axis in Figure 4. The structural differences between the BCC Cu agglomerate and the matrix could be accommodated by dislocations in a semi-coherent interface.

### 3.4. Morphology Transition to Porous VCM Caused by Increasing Temperature

**Figure 5:**
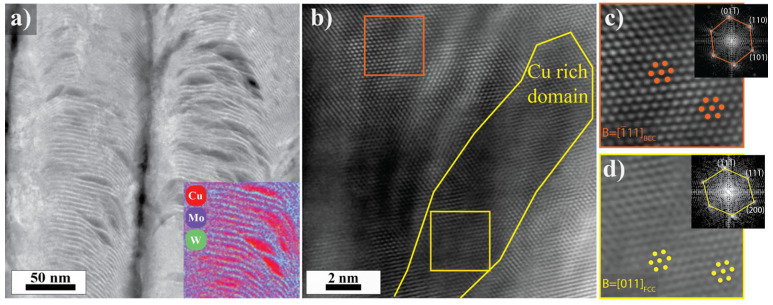
(**a**) HAADF-STEM and STEM-EDS of co-sputtered Cu-Mo-W deposited at 800 °C displaying a heterogeneously phase separated morphology with a VCM matrix and Cu agglomerates. (**b**) HRSTEM showing the coherent BCC matrix and FCC Cu domain. (**c**) The FFT of the BCC matrix is along the $\left[\bar{1}11\right]$ zone axis. (**d**) The FFT of the Cu-rich domain is indexed as the [011] FCC zone axis.

HAADF-STEM and STEM-EDS of the sample deposited at 800 °C are presented in Figure 5a. Columnar film growth morphology with some intercolumnar porosity is evident. Columnar grains are 50–100 nm in diameter. The porosity is accentuated by the FIB preparation of the TEM lamella.

HAADF-STEM shows that each grain in the 800 °C sample has a vertical concentration modulation (VCM) in which the layers are oriented perpendicular to the growing direction at the centerline of each grain. Toward the edge of each grain, the concentration modulation becomes inclined, mimicking the surface topology of each grain’s curved surfaces. The concentration modulations are composed of alternating Cu layers and Mo-W solid solution layers, and the concentration modulation is disrupted by agglomerates of the Cu-rich phase, as seen by the STEM-EDS inset of Figure 5a. Multiple length scales must be used to characterize the morphology of the 800 °C sample, so the morphology can be classified as heterogeneous. The VCM bilayer wavelength is 5 nm along the center line of the grains.

An HRSTEM micrograph of the 800 °C sample is presented in Figure 5b. A Cu agglomerate is marked in yellow and is surrounded by the bicontinuous matrix. The matrix has a BCC structure. The Cu and Mo-W layers are coherent, and Cu has a pseudomorphic structure. The Cu agglomerates have the equilibrium FCC structure and are heavily faulted to remain partially-coherent with the surrounding matrix. Figure 5c shows the BCC matrix oriented along the $\left[\bar{1}11\right]$ zone axis and Figure 5d shows the FCC Cu agglomerates oriented along the [011] zone axis.

### 3.5. Hardness of Heterogeneous Cu-Mo-W Thin Films

**Figure 6:**
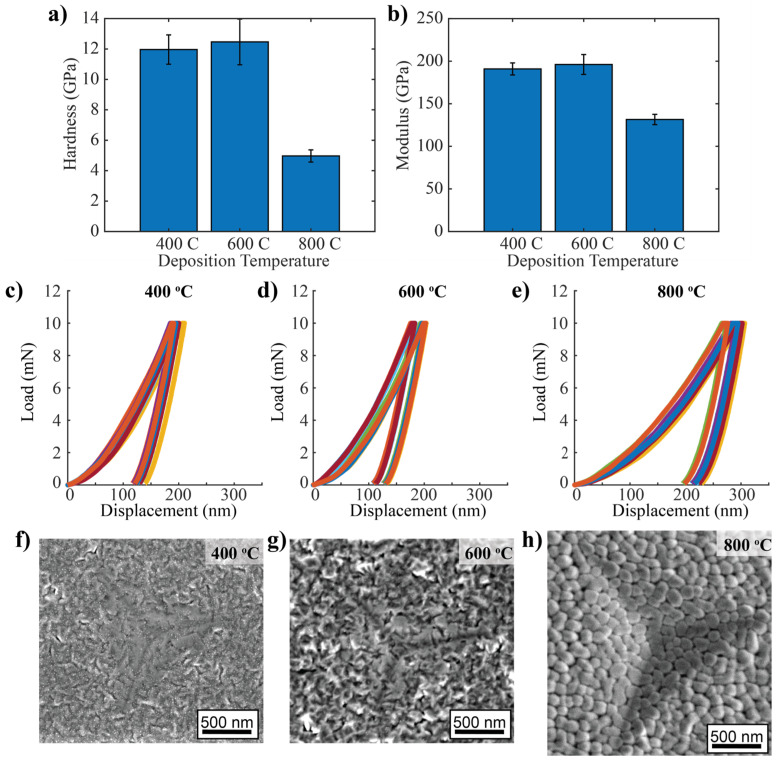
A comparison of the (**a**) hardness and (**b**) reduced modulus of the three nanocomposites from this study. Load-displacement curves of the 9 indents for the (**c**) 400 °C, (**d**) 600 °C and (**e**) 800 °C samples. Each indent was performed with the same conditions and the colors are only used to visually differentiate each curve. Secondary electron SEM images showing the surface topology and indents for the (**f**) 400 °C, (**g**) 600 °C and (**h**) 800 °C samples.

The average hardness of the 400 °C, 600 °C, and 800 °C samples were 12.0 ± 1.0, 12.5 ± 1.5, and 5.0 ± 0.4 GPa, respectively, as shown in Figure 6a. The average reduced moduli were 190.9 ± 7.1, 196.1 ± 11.7, and 131.5 ± 6.0 GPa, respectively, as shown in Figure 6b. The averages were calculated from 9 indents, the load-displacement profiles of which are presented in Figure 6c–e. SEM images of the indents do not reveal any obvious pile-up or shear localization, as shown in Figure 6f–h. The indents cover tens of columnar grains, and the pores are orders of magnitude smaller than the indent size, so the measured indentation hardness and moduli are representative of these microstructures. In other words, even though the microstructures are heterogeneous, their hardness appears homogeneous because of the smaller length scales of the microstructural features as compared to size of the indent.

## 4. Discussion

The motivation for this work was to explore how the addition of ternary alloying elements to binary phase separating systems affected morphological evolution in growing co-sputtered films as a function of deposition temperature and deposition rate. W was added to the model Cu-Mo binary system because W increases the difference in surface interdiffusion length [7]. The addition of W led to morphologies that are indicative of shortened surface interdiffusion lengths but also introduced unexpected features such as Mo-W rich agglomerates. The resulting microstructures of the samples deposited at 400 °C and 600 °C had heterogeneous morphologies consisting of bicontinuous CMs and clusters of Mo-W rich agglomerates. These morphologies are unique, since the agglomerates are composed of the slower-diffusing species, Mo-W. The nanoindentation results are consistent with high hardness due to nano-confinement of dislocation slip and concentrated agglomerates of nanoscale BCC refractory metal solid solutions.

### 4.1. Microstructural Effect of Alloying with W

Alloying the model Cu-Mo system with W via co-sputtering led to multiple microstructural changes indicative of inhibited diffusion which are explained in detail below.

#### 4.1.1. Fine Concentration Modulations Which Persist up to 800 °C

The bicontinuous CMs in the Cu-Mo-W films deposited at 400 °C and 600 °C had a bilayer wavelength of approximately 3 nm. While there was a large change in the phase separated morphology when the deposition temperature was raised to 800 °C, the bilayer wavelength remained very fine at approximately 5 nm. In comparison, Cu-Mo films deposited at a rate of 1.4 nm/s had CM wavelengths of 10 nm, 16 nm, and 32 nm for the 400, 600, and 800 °C depositions, respectively [6]. A faster deposition rate should have limited the surface interdiffusion distance, but the Cu-Mo nanocomposites still had larger bilayer wavelengths. At a lower deposition rate of 0.12 nm/s, Cu-Mo deposited at 800 °C had a 72 nm bilayer wavelength [10]. Cu-Mo-W has finer CMs than Cu-Mo, which indicates that the addition of W reduces the surface interdiffusion length of the adatoms.

#### 4.1.2. Pseudomorphic Cu in the Matrix at Higher Temperatures

Pseudomorphic Cu in the matrix is an indicator of small layer thicknesses or the early stages of spinodal decomposition [17,18]. For Cu-Mo co-deposited at 1.4 nm/s, processing temperatures of up to 400 °C resulted in pseudomorphic Cu. FCC Cu appeared at 500 °C. However, the Cu-Mo-W had pseudomorphic Cu in the matrix up to 800 °C. This shows that the addition of W inhibits the complete phase separation of the Cu-Mo-W mixture at the surface before each layer is buried by the incoming deposition flux.

#### 4.1.3. VCM Morphology at High Temperature

A VCM structure is indicative of incomplete phase separation at the growing surface [9]. A transition from a VCM to an LCM structure was observed between 400 and 600 °C in co-sputtered Cu-Mo at 1.4 nm/s. Surface diffusion in binary Cu-Mo increases enough at 600 °C to completely phase separate at the surface. However, the mixture of Cu-Mo-W does not phase separate completely at the surface, even though the deposition rate is lower and there is more time to diffuse. This is another indication that W impedes surface diffusion.

#### 4.1.4. Porous Columnar Film at High Temperature

Structural zone diagrams classify thin film structures by surface morphology and grain shape [19,20]. For pure and dilute alloy thin films, transitions between the zones can be caused by increasing the deposition temperature. Zone 1 consists of porous, tapered crystallites and occurs when $T_{h}<0.3$. Zone 2 has columnar grains with closed pores and occurs when $0.3<T_{h}<0.45$. Zone T is a transition zone that develops at temperatures between Zone 1 and Zone 2. Finally, Zone 3 consists of recrystallized grains and occurs when $T_{h}>0.45$ [19,20].

The film deposited at 800 °C is highly porous, as seen in the plan view SEM in Figure 6e and the cross-sectional STEM in Figure 5a. This is consistent with Zone 1 or Zone T. The $T_{h}$ values for Mo and W are 0.37 and 0.29, respectively, so the porous structure fits reasonably well with the predictions of the structural zone diagrams. The formation mechanism for Zone 1 and Zone T is called shadowing. Surface roughness in the early stages of film growth is accentuated, because high points block low points from the deposition flux [21]. Low $T_{h}$ causes slow surface diffusion, so diffusion cannot fill the pores as quickly as they form. Therefore, the porous structure of the sample deposited at 800 °C is indicative of slow surface diffusion caused by alloying with W.

The films deposited at 400 and 600 °C were less porous, as seen in the cross-sectional TEM micrographs in Figure 2a and Figure 3a, and in the plan-view SEM micrographs in Figure 6c,d. Densification is expected with increasing temperature, so this finding is unexpected. However, structural zone diagrams were developed with pure and dilute alloys in mind [19,20]. The highly disparate melting temperatures and surface diffusivity of the immiscible components in these alloys may lead to deviations from structural zone diagram trends.

### 4.2. Agglomerate Cluster Formation Mechanism

The heterogeneous structures reported for the 400 °C and 600 °C samples in this study are the first co-sputtered immiscible thin films to have a bicontinuous matrix and agglomerates composed of the slower-diffusing species. This is a unique feature, because previous studies attributed agglomerate formation to quick surface diffusion and island type growth. Powers et al. investigated the formation mechanism of heterogeneous immiscible nanocomposites using phase field modeling and an experimental investigation of binary immiscible nanocomposites [11]. The phase field models showed that local fluctuations in the deposition flux composition could lead to heterogeneous morphologies. In the model, regions with greater than 70% Cu locally formed Cu-rich agglomerates with trapped Mo.

In Cu-Mo-W, the Cu in the agglomerate clusters forms veins running parallel to the surface rather than nanoprecipitates. One possible explanation for this morphology is an extension of the explanation for VCM formation put forward by Lu et al. [9]. During growth, the total interfacial energy is composed of contributions from the BCC-FCC, BCC-vacuum, and FCC-vacuum interfaces. The mixed surface layer phase separates so that the composition at the bottom of the surface layer matches the composition of the subsurface layer. This effectively reduces the amount of BCC-FCC surface area. Even though spheres have lower surface energy and would lead to the lowest interfacial energy in the final microstructure when only BCC-FCC interfaces are considered, planar agglomerates minimize interfacial energy during growth, when interfaces with vacuum are also important. The Cu veins are kinetically frozen into the final microstructure.

### 4.3. Dependence of Hardness on Tortuosity in Heterogeneous Cu-Mo-W Thin Films

Immiscible nanocomposites derive their strength from multiple factors. This study is mostly concerned about the effects of the heterogeneous phase separated structure within the columnar grains, but the effects of porosity and composition also effect the strength.

The sample deposited at 800 °C had a much lower hardness and modulus compared to the other samples. While the slight increase in the bilayer wavelength and the increased Cu composition also contribute to the lower hardness, porosity is the main cause of this change in properties. The intercolumnar porosity of the 800 °C sample can be seen in cross-section in Figure 5 and in plan-view in Figure 6. The VCMs within each column are discontinuous across columns. Monclus et al. studied Cu-W multilayers, which also showed a columnar structure and discontinuous layers [22]. While reducing the layer thickness is expected to increase strength, Monclus did not observe a dependence of hardness on layer thickness. The hardnesses ranged from 4.9 GPa to 5.5 GPa, and the Cu-Mo-W samples in this study had a hardness of 5.0 ± 0.4 GPa.

**Figure 7:**
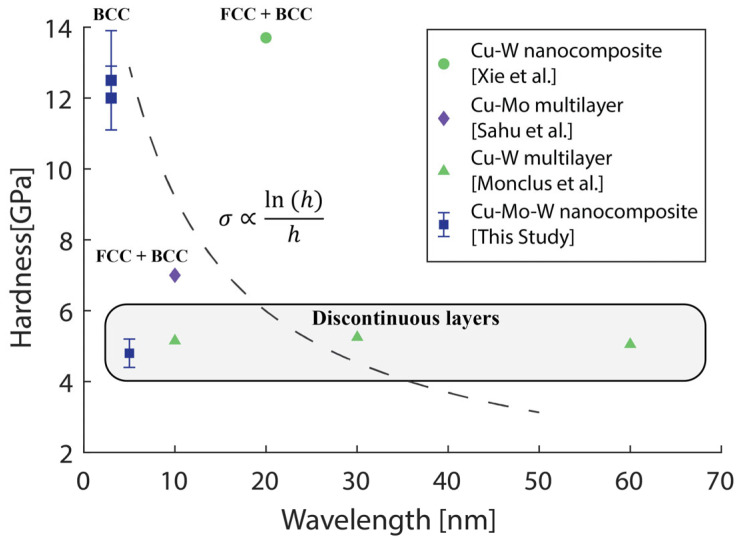
A comparison of hardness as a function of bilayer wavelength for immiscible nanocomposites containing Cu-Mo or Cu-W layers [22,23,24]. The CLS strengthening trend is shown by the dashed line. Deviations from the CLS trend arise due to confounding factors such as porosity and composition differences.

The high strengths of the heterogeneous Cu-Mo-W nanocomposites deposited at 400 and 600 °C are of particular interest. These nanocomposites have some of smallest bilayer thicknesses reported for immiscible nanocomposites, and they contain a new agglomerate structure for heterogeneous nanocomposites. The closest comparisons to these samples are binary Cu-Mo or Cu-W nanocomposites and multilayers. Figure 7 shows a comparison between the Cu-Mo-W nanocomposites and the binary nanocomposites that have hardnesses reported in literature.

One high strength Cu-W nanocomposite reported in literature had an indentation hardness of 13.8 GPa. The bilayer wavelength was reported to be 20 nm, and there were FCC Cu layers and BCC W layers [23]. One high strength Cu-Mo multilayer reported in the literature had an indentation hardness of 8 GPa [24]. The bilayer wavelength was reported to be 10 nm, and it also had FCC Cu and BCC Mo layers. These thin films do not follow the CLS strengthening trend, σ=ln(h)/h, marked by the dashed line in Figure 7, because of the difference in hardness between Mo and W and the different composition of each thin film.

The Cu-Mo-W nanocomposite has a strength intermediate to the binary comparisons. The fine bicontinuous microstructure is still responsible for much of the strengthening of the Cu-Mo-W nanocomposite—the hardnesses of pure, single crystal Cu, Mo, and W are much lower at 0.27, 1.5, and 3.6 GPa, respectively [25,26]. The increase in hardness compared to the Cu-Mo multilayer with a 10 nm bilayer wavelength is caused by a mixture of further layer refinement and replacing a fraction of the Mo with W.

There are few reports on the mechanical properties of other heterogeneous thin films. The Cu-Mo system has received the most extensive characterization, but hardness comparisons are confounded by the different compositions as well as dissimilar heterogeneous structures. The Cu-Mo heterogeneous nanocomposites had Cu agglomerates [10]. The inclusion of the Cu agglomerates barely reduced the hardness. A multilayer with a 3 nm bilayer wavelength had a flow stress of 2.6 GPa while a heterogeneous nanocomposite with a 3 nm bilayer wavelength and 200 nm Cu agglomerates maintained a flow stress of 2.6 GPa for a micropillar compression experiment [12].

The heterogenous Cu-Mo-W nanocomposite is not expected to exhibit such an increase in plastic deformability because the Mo-W agglomerate cannot deform plastically to the same degree as Cu agglomerates. At room temperature, single crystal Mo micropillars failed at less than 2% engineering strain, and micro-architecture W coatings that underwent micropillar compression at room temperature had limited strain hardening capacity compared to those compressed at elevated temperatures [27,28]. Therefore, it is not expected that plastic deformability will improve significantly through co-deformation.

## 5. Conclusions

The addition of W to the co-sputtered immiscible Cu-Mo system impeded diffusion, leading to finer concentration modulations and pseudomorphic Cu layers at temperatures of up to 800 °C. The co-sputtered Cu-Mo-W nanocomposites deposited at 400 °C and 600 °C have a novel heterogeneous structure that includes a bicontinuous matrix and Mo-W agglomerate clusters. The bicontinuous matrix is a common feature shared with Cu-Mo nanocomposites, but the Mo-W agglomerate clusters are unique because the agglomerate is composed primarily of the slower diffusion species. While phase field modeling and model binary systems have begun to provide insight into the formation mechanism of co-sputtered immiscible nanocomposites, the complexity of the phase separation during phase growth, especially when high temperatures, low deposition rates, and large differences in mobilities between species come into play, allows for novel structures only accessible by experimentation.

Nanoindentation shows a high hardness of 12–12.5 GPa for the Cu-Mo-W nanocomposites, but due to the lack of a soft phase to co-deform with the bicontinuous matrix, the improvements in plastic deformability are expected to be limited compared to Cu-Mo. Regardless, this study demonstrates how W modified the heterogenous composite structure due to its low mobility. The consequence of the ultrafine bicontinuous matrix is high hardness. BCC refractory metal thin films have applications as coatings in high-temperature environments, such as heat exchangers in nuclear reactors or gas turbines, but their poor plastic deformability at room temperature is a critical issue. While the heterogeneous Cu-Mo-W nanocomposites in this study did not ultimately produce morphologies that are expected to improve plastic deformability by co-deformation, the insights gained about processing heterogeneous thin films with high melting point refractory metals may help guide future research about using heterogeneous microstructures to control the plastic deformability of refractory metal thin films.

## Figures and Tables

**Table 1:** Deposition temperature, composition, and deposition rate of the three co-sputtered Cu-Mo-W samples from this study.

Deposition Temperature	Composition Cu-Mo-W (at.%)	Combined Deposition Rate
400 °C	40-31-29	0.28 nm/s
600 °C	41-29-30	0.28 nm/s
800 °C	47-29-24	0.27 nm/s

## Data Availability

The data presented in this study are openly available in Materials Commons at https://doi.org/10.13011/m3-g0xj-5789.
